# The joint-local renin–angiotensin system in rheumatoid arthritis and osteoarthritis: mechanistic evidence, disease-specific patterns, and translational perspectives

**DOI:** 10.1007/s00296-026-06073-9

**Published:** 2026-02-05

**Authors:** Emre Bilgin, İbrahim C. Haznedaroğlu

**Affiliations:** 1 Division of Rheumatology, Department of Internal Medicine, Faculty of Medicine, Sakarya University, Sakarya, Turkey; 2 Division of Hematology, Department of Internal Medicine, Faculty of Medicine, Hacettepe University, Ankara, Turkey

**Keywords:** Renin-angiotensin system, Rheumatoid arthritis, Osteoarthritis, Synovial membrane, Angiotensin-converting enzyme

## Abstract

The renin–angiotensin system (RAS), traditionally regarded as a hormonal cascade regulating cardiovascular and renal homeostasis, is increasingly recognized as a locally active, tissue-specific network within joint structures. Accumulating evidence indicates that synovial tissue, synovial fluid, and articular cartilage harbor a functionally active joint-local renin-angiotensin system that operates partially autonomously from the systemic RAS circulation and is implicated in the pathogenesis of both arthritis (RA) and osteoarthritis (OA). This narrative review integrates human, animal, and in vitro evidence to examine the dual-axis organization of the joint RAS, comprising a pathogenic angiotensin-converting enzyme (ACE)/Angiotensin II (Ang II)/ Angiotensin II type 1 receptor (AT1R) axis and a counter-regulatory ACE2/Angiotensin-(1–7) (Ang-(1–7))/Mas receptor-Mas related G protein-coupled receptor D (Mas–MrgD) axis, and to explore how imbalance between these pathways may differentially influence inflammatory and degenerative joint diseases. In RA, experimental and translational studies suggest that enhanced activity of the classical axis within synovial tissue is associated with synovial inflammation, fibroblast-like synoviocyte survival, angiogenesis, and bone erosion through pathways involving nuclear factor kappa B (NF-kB), mitogen-activated protein kinase (MAPK), and receptor activator of nuclear factor kB ligand (RANKL)/Wingless-related integration site (Wnt) signaling pathway. In OA, available data indicate that chondrocyte expression of AT1R/Angiotensin II type 2 receptor (AT2R), together with cytokine-induced receptor upregulation, may sensitize cartilage to Ang II-mediated effects, contributing to matrix metalloproteinase-13 (MMP-13)–mediated matrix degradation and activation of interleukin-6 (IL-6)/janus kinase 2 (JAK2)/signal transducer and activator of transcription 3 (STAT3) signaling. Genetic studies support disease-specific patterns, with the ACE insertion/deletion polymorphism showing a more consistent association with RA susceptibility than with knee OA, although findings vary across populations and do not consistently correlate with disease severity. From a therapeutic perspective, modulation of the joint-local RAS is currently supported mainly by preclinical evidence. Experimental models suggest that classical RAS inhibitors and emerging strategies targeting the protective axis—such as putative ACE2 activators, AT2R agonists, and bone-targeted peptide delivery can influence inflammatory and structural pathways within the joint, while direct clinical evidence remains limited. Overall, current data support the biological relevance of a local joint RAS in arthritis pathophysiology and highlight key gaps between experimental findings and clinical translation.

## Introduction: from systemic regulator to local joint modulator

The Renin-Angiotensin System (RAS) has long been recognized as a vital hormonal cascade responsible for maintaining cardiovascular (CV) and renal homeostasis. Within this framework, the RAS regulates blood pressure, fluid volume, and electrolyte balance through a coordinated endocrine mechanism [1]. In recent decades, this systemic view has expanded significantly. Evidence now shows that many tissues harbor a complete, locally contained, and functionally independent RAS, collectively referred to as tissue-specific renin-angiotensin systems (tRAS) [2]. Components of tRAS are produced and act locally in organs such as the liver, heart, lung, and, critically for this review, in bone and articular structures.

The skeletal RAS has emerged as a direct and significant regulator of bone metabolism [3]. Concurrently, accumulating evidence shows that the joint—including the synovium, synovial fluid, and chondrocytes—is a site of a functionally active, locally regulated RAS [4]. This local joint RAS is increasingly implicated as an active part of the inflammatory processes, tissue remodeling, and angiogenic pathways that characterize major joint diseases [3, 4].

In recent years, the RAS has been re-defined not only as a hormonal cascade but also as an immunometabolic network active within inflamed tissues [5]. Angiotensin II (Ang II) stimulates macrophages, dendritic cells, and T cells through the angiotensin II type 1 receptor (AT1R), mainly by increasing oxidative stress and nuclear factor kappa B (NF-kB) signaling. In contrast, the angiotensin-converting enzyme 2 (ACE2)/Angiotensin-(1–7)(Ang-(1–7))/Mas receptor (MasR) axis promotes macrophage M2 polarization and suppresses inflammasome activation [6]. This immune-oriented view helps explain why RAS behaves differently in autoimmune diseases such as rheumatoid arthritis (RA) compared with mechanically and metabolically driven osteoarthritis (OA).

This review aims to critically analyze and synthesize current evidence to address four key questions: (1) the components and dual-axis nature of the local joint RAS; (2) the distinct pathogenic roles of this system in RA and OA; (3) the contribution of genetic factors, particularly the ACE insertion/deletion (I/D) polymorphism to disease susceptibility; and 4) the preclinical and clinical evidence supporting therapeutic modulation of the joint RAS.

Several recent reviews have discussed the role of the RAS in RA and OA from broader systemic or comorbidity-oriented perspectives [7, 8]. To clarify the specific scope and focus of the present review, its main scope and perspectives are outlined in Table 1.

**Table 1:** Scope and perspective of the present review

Focus of contribution	Primary focus and approach	Related sections
Joint as the primary unit of analysis	The review treats the joint as the main biological context and examines RAS activity based on findings from synovial tissue, synovial fluid, cartilage, and subchondral bone.	Sections “The Dual-Axis Paradigm of the RAS”–“The Local RAS in OA Pathogenesis”
Mechanistic separation of RA and OA at the joint level	RA and OA are discussed as distinct joint diseases, with emphasis on differences in synovial inflammation, cartilage responses, and local RAS signaling.	Sections “The Local RAS in RA Pathology” and “The Local RAS in OA Pathogenesis”
Joint-centered alignment of evidence layers	Human joint data, animal models, and in vitro findings are discussed together within a unified mechanistic framework, allowing differences in evidence strength to be seen clearly.	Section “The Counter-Regulatory (Protective) Axis”–“The Role of the AT2R”, “Evidence for an Active and Independent Synovial RAS”–“RAS-Mediated Angiogenesis and Bone Destruction”
Local interpretation of genetic findings	Genetic associations involving RAS components are contextualized with respect to joint pathology and disease phenotype, with attention to heterogeneity and limits of clinical inference.	Section “Genetic Predisposition: The ACE Insertion/Deletion (I/D) Polymorphism”
Conceptual link between local RAS regulation and JAK inhibition	The review introduces a joint-level perspective on how JAK inhibitors and other DMARDs may influence local RAS balance, framed as a mechanistic hypothesis.	Section “Therapeutic Targeting of the Local Joint RAS”
Future research directions grounded in joint biology	The review outlines future research questions arising from a joint-local RAS perspective, emphasizing the need for prospective study designs, tissue-specific measurements, and mechanistic validation rather than clinical recommendations.	Section “Conclusion and Future Directions”

DMARD, disease-modifying antirheumatic drug; JAK, Janus kinase; OA, osteoarthritis; RA, rheumatoid arthritis; RAS, renin–angiotensin system

## Methods

This article is a narrative, non-systematic review, conducted in accordance with published recommendations for narrative biomedical reviews [9].

. A literature search was conducted in PubMed, Scopus, and Web of Science, covering publications from database inception through September 2025 to identify studies examining the role of the RAS in joint-related pathology, with a specific focus on RA and OA.

Searches were performed using combinations of keywords related to both RAS components and joint disease. An example search strategy used in PubMed was:

(“renin–angiotensin system” OR angiotensin OR ACE OR ACE2) AND (rheumatoid arthritis OR osteoarthritis) AND (synovium OR synovial fluid OR cartilage). Comparable search terms were adapted for use in Scopus and Web of Science.

Studies were considered eligible if they investigated RAS components in joint-derived tissues or fluids, including synovial tissue, synovial fluid, articular cartilage, or subchondral bone. Both human studies and experimental research (animal models and in vitro studies) were included. Studies focusing exclusively on systemic, cardiovascular, renal, or circulating RAS markers without direct relevance to joint biology were not included as core evidence. Review articles were used selectively to provide background context, but were not treated as primary sources of evidence.

Consistent with the narrative nature of the review, no formal protocol, predefined quality scoring, or quantitative synthesis was applied. Greater weight was given to studies that provided direct tissue-level data, mechanistic insight into joint pathology, or links between experimental findings with clinical observations. Evidence was assessed qualitatively, with attention to biological plausibility, consistency across different study types, and relevance to joint-specific RAS regulation, in line with established guidance for narrative biomedical reviews [9].

## The dual-axis paradigm of the RAS

**Fig. 1:**
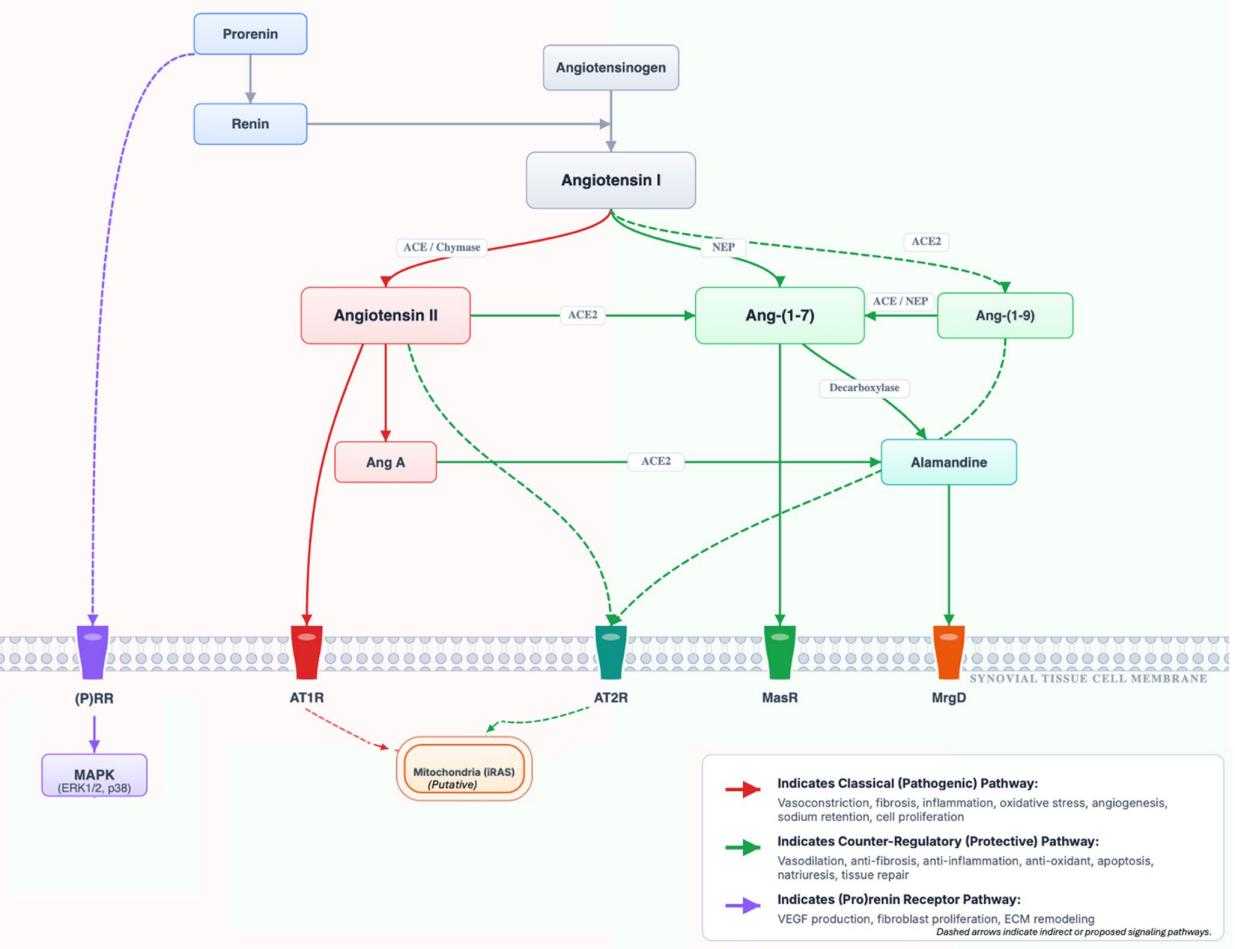
Overview of joint-local renin–angiotensin system pathways and receptor signaling. ACE, angiotensin-converting enzyme; ACE2, angiotensin-converting enzyme 2; Ang A, angiotensin A; Ang I, angiotensin I; Ang II, angiotensin II; Ang-(1–7), angiotensin-(1–7); Ang-(1–9), angiotensin-(1–9); AT1R, angiotensin II type 1 receptor; AT2R, angiotensin II type 2 receptor; ECM, extracellular matrix; ERK1/2, Extracellular signal-Regulated Kinase 1 and 2; iRAS, intracellular renin–angiotensin system; MAPK, mitogen-activated protein kinase; MasR, Mas receptor; MrgD, Mas-related G protein-coupled receptor D; NEP, neprilysin; (P)RR, (pro)renin receptor. This figure is an original schematic prepared by the authors based on published literature

The modern understanding of the RAS is that it involves a dynamic balance between two opposing axes. The pathological result of RAS activation in tissues, including the joint, can be seen as an imbalance or “tipping of the scales,” where the activity of a pathogenic axis surpasses that of a protective, counter-regulatory axis [10]. A schematic representation of this proposed network, illustrating the interactions between classical and counter-regulatory axes within the joint, is presented in Fig. 1, integrating evidence from experimental and human studies [1, 5, 11].

### The classical (pathogenic) axis

The classical RAS axis consists of ACE, which converts Angiotensin I (Ang I) into the main effector peptide Ang II [11]. Ang II mediates most of its well-known effects by binding to the AT1R [11]. In tissue pathology, activation of the ACE/Ang II/AT1R axis has particularly pathogenic, pro-inflammatory, pro-oxidative, and pro-fibrotic effects while promoting vasoconstriction and angiogenesis [11]. Within the joint, this axis is hypothesized to be the major contributor to RAS-mediated inflammation and tissue damage [4].

In addition to ACE-dependent Ang II formation, mast cell–derived chymase provides an ACE-independent serine-protease pathway that converts Ang I directly to Ang II [12]. In inflamed synovial tissue, where mast cell numbers and chymase levels are increased, this alternative pathway may sustain local Ang II generation despite ACE inhibition [13, 14]. Chymase-driven Ang II formation is suspected to amplify local RAS activity in RA synovium and may contribute to ACE-inhibitor “RAS escape” at the joint level [15].

The (pro)renin receptor (PRR) activates extracellular signal-regulated kinases 1/2 (ERK1/2) and p38 mitogen-activated protein kinase (MAPK) through a renin-enzymatic-activity–independent mechanism, acting as a signaling receptor rather than only a cofactor in angiotensin generation [16].

PRR expression has been demonstrated in endothelial and synovial tissues, suggesting that it may contribute to enhanced local RAS activity even when circulating renin concentrations are low [17].

Through downstream MAPK activation, PRR has been shown to increase angiogenic signalling such as vascular endothelial growth factor (VEGF) production, support fibroblast proliferation, and modulate extracellular matrix turnover [18]. Although its direct contribution to RA pathogenesis has not been fully delineated, these renin-independent effects provide a biologically plausible mechanism by which PRR signaling could amplify chronic inflammation, angiogenesis, and structural remodeling characteristic of pannus formation.

### The counter-regulatory (protective) axis

The counter-regulatory axis acts as an intrinsic brake on the classical pathway [5]. This axis is centered on ACE2, an enzyme that converts the pathogenic Ang II into a new peptide, Ang-(1–7). Ang-(1–7) then signals through its own receptor, the MasR [5]. The actions of this ACE2/Ang-(1–7)/MasR axis are functionally antagonistic to those of the classical axis. It exerts potent anti-inflammatory, anti-fibrotic, and anti-proliferative effects, while also promoting vasodilation and overall tissue protection [5].

Ang-(1–7) can also be synthesized independently of ACE2 through neprilysin (NEP, also known as cluster of differentiation 10 (CD10))-mediated conversion of Ang I [19]. In arthritic joints, NEP/CD10 is detectable in synovial tissue and fluid [20]. It may act as a compensatory mechanism that both degrades pro-inflammatory neuropeptides and supports the protective Ang-(1–7)–MasR axis. However, its precise regulation in RA versus OA tissues remains incompletely defined. Beyond generating Ang-(1–7), ACE2 can also convert Ang I into Angiotensin-(1–9)(Ang-(1–9)), another peptide with anti-fibrotic and anti-apoptotic functions via the angiotensin II type 2 receptor (AT2R). Loss of ACE2 in inflamed joints may therefore reduce two separate protective ligands simultaneously.

Beyond the ACE2/Ang-(1–7)/MasR pathway, the non-classical RAS also includes alamandine, a heptapeptide generated from Ang-(1–7) or Angiotensin A, which signals via the Mas-related G-protein-coupled receptor D (MrgD) [21]. Alamandine reproduces many of the vasodilatory, anti-fibrotic and anti-hypertensive actions of Ang-(1–7) while using a distinct receptor, suggesting that it may preserve protective signaling even when Mas expression is downregulated [22]. This axis has been implicated in CV and fibrotic disease models and conceptually extends the protective arm of the RAS that could also operate in inflamed joint tissues [23].

Together, ACE2-derived peptides—Ang-(1–7) and Ang-(1–9)—form a coherent counter-regulatory network that blunts AT1R signaling, limits oxidative stress and reduces fibroblast proliferation [5, 24]. The coexistence of NEP/CD10-dependent Ang-(1–7) production in synovium suggests that several parallel protective routes operate in joints, although their relative contribution appears disease-dependent [20].

### Intracellular and mitochondrial RAS (iRAS)

Ang II signaling is not limited to the cell surface. Internalized angiotensin receptors and locally generated peptides form a mitochondrial and intracellular renin-angiotensin system (iRAS) that regulates oxidative phosphorylation, reactive oxygen species (ROS) production and cell survival [25]. In mitochondria, Ang II and AT2R/AT1R influence nitric oxide (NO) production and respiratory control, providing a direct link between RAS and redox homeostasis [25]. Although most mechanistic data derive from CV and renal tissues, these findings may support a model in which intracellular RAS in synovial fibroblasts could sustain oxidative stress and proliferation even when extracellular AT1R is pharmacologically blocked. Direct evidence for iRAS involvement in synovial fibroblasts is currently lacking.

### The role of the AT2R

Although AT2R signaling is often grouped conceptually with counter-regulatory mechanisms, it differs from the ACE2/Ang-(1–7)/MasR axis in that it is activated by Ang II itself and operates within the classical ligand framework [6]. While it also binds the pathogenic Ang II peptide, its downstream effects generally oppose those of the AT1R [6]. AT2R signaling is mostly considered protective, mediating anti-inflammatory, anti-fibrotic, and pro-apoptotic effects that counteract AT1R-driven proliferation [6].

AT1R activation stimulates Gq/11–protein kinase C (PKC), ROS generation, and MAPK (ERK/c-Jun N-terminal kinase (JNK)/p38), all of which amplify inflammation and fibroblast proliferation [26]. In contrast, AT2R activates phosphatases such as protein phosphatase 2 A (PP2A), enhances NO–cyclic guanosine monophosphate (cGMP) signaling, and blunts ERK1/2 activity, resulting in anti-proliferative and anti-fibrotic effects [24]. The functional imbalance between AT1R-dominant and AT2R-dominant signaling, therefore, determines whether tissue homeostasis is restored or chronic fibroinflammatory responses persist.

The relative activation of these opposing axes within the joint microenvironment seems to be a key factor in determining joint health and disease, as illustrated by the comparative effects detailed in Table 2.

**Table 2:** Components and functions of the Dual-Axis joint Renin-Angiotensin system

Component	Key player	Primary role in articular pathophysiology
*Pathogenic Axis*		
Enzyme	Angiotensin-Converting Enzyme (ACE)	Produces Ang II, which promotes the pathogenic cascade.
Peptide	Angiotensin II (Ang II)	Binds AT1R/AT2R. Activates pro-inflammatory mediators, oxidative stress, and fibrosis.
Receptor	Angiotensin II Type 1 Receptor (AT1R)	Mediates most pathogenic effects of Ang II: inflammation, FLS proliferation, angiogenesis, and bone resorption.
Enzyme	Chymase (mast cell–derived)	ACE-independent pathway converting Ang I → Ang II. Elevated in inflamed synovium; sustains Ang II production despite ACE inhibition and contributes to RAS escape.
Receptor	(Pro)renin Receptor (PRR)	Activates ERK1/2, p38 MAPK independent of renin activity; promotes VEGF, fibroblast proliferation, and ECM remodeling; may sustain local Ang II activity even when systemic renin is low.
*Counter-Regulatory (Protective) Axis*		
Enzyme	Angiotensin-Converting Enzyme 2 (ACE2)	Degrades Ang II to Ang-(1–7), decreasing classical axis activity and enhancing protective axis.
Enzyme	Neprilysin (NEP/CD10)	Produces Ang-(1–7) from Ang I, degrades pro-inflammatory neuropeptides; supports ACE2/Mas protective axis in synovium.
Peptide	Angiotensin-(1–7)	Binds MasR. Shows anti-inflammatory, anti-fibrotic, and tissue-protective effects.
Peptide	Angiotensin-(1–9)	Generated by ACE2 from Ang I; binds AT2R; anti-fibrotic, anti-apoptotic, protective. Putative protective role based on cardiac models; joint-specific data are limited.
Peptide	Alamandine	Ang-(1–7)– or Ang A–derived protective peptide; vasodilatory, anti-fibrotic, anti-hypertensive; complements Mas axis, especially when MasR is downregulated.
Receptor	Mas Receptor (MasR)	Mediates the protective effects of Ang-(1–7), reducing cytokine release and tissue damage.
Receptor	Mas-related G-protein-coupled receptor D (MrgD)	Receptor for alamandine; mediates anti-fibrotic and vasoprotective effects; part of extended protective RAS arm.
*Intracellular / Mitochondrial Axis (iRAS)*		
Intracellular RAS	Internalized AT1R/AT2R + locally produced Ang II in mitochondria	Regulates mitochondrial ROS, oxidative phosphorylation, NO generation; sustains fibroblast proliferation even when extracellular AT1R blocked.
*Modulatory Receptor*		
Receptor	Angiotensin II Type 2 Receptor (AT2R)	Activates phosphatases (PP2A), increases NO–cGMP signaling, reduces ERK1/2 activity; anti-proliferative/anti-fibrotic.

ACE = Angiotensin-converting enzyme; ACE2 = Angiotensin-converting enzyme 2; Ang I = Angiotensin I; Ang II = Angiotensin II; Ang-(1–7) = Angiotensin-(1–7); Ang-(1–9) = Angiotensin-(1–9); AT1R = Angiotensin II type-1 receptor; AT2R = Angiotensin II type-2 receptor; CD10 = Cluster of differentiation 10 (neprilysin); ECM = Extracellular matrix; ERK1/2 = Extracellular signal-regulated kinase 1/2; FLS = Fibroblast-like synoviocyte; iRAS = Intracellular renin–angiotensin system; MAPK = Mitogen-activated protein kinase; MasR = Mas receptor; MrgD = Mas-related G-protein-coupled receptor D; NEP = Neprilysin; NO = Nitric oxide; PP2A = Protein phosphatase 2 A; PRR = (Pro)renin receptor; RAS = Renin–angiotensin system; ROS = Reactive oxygen species; VEGF = Vascular endothelial growth factor

## The local RAS in RA pathology

In RA, the local joint RAS is not only present but appears to function as a hyperactive, locally amplified, and partially autonomous system implicated in synovial inflammation, pannus formation, and bone erosion [4, 7].

### Evidence for an active and independent synovial RAS

Seminal studies have provided direct evidence of an active RAS within the RA joint. Çobankara et al., in a foundational study published in *Rheumatology International*, measured active renin and ACE levels in both serum and synovial fluid (SF) from patients with RA, OA, and healthy controls [27]. While serum levels of these components were comparable across all groups, the concentrations of both ACE and active renin were significantly higher in the synovial fluid of RA patients compared to OA patients [27].

This finding has been confirmed in other studies. Wu et al. used multiple complementary techniques (enzyme-linked immunosorbent assay [ELISA], quantitative polymerase chain reaction [qPCR], Western blot, and immunohistochemistry) to analyze synovial fluid and tissue [28]. Their results showed that the protein and mRNA expression levels of renin, ACE, AT1R, and AT2R were all significantly higher in RA patients compared to OA patients [28].

These data reveal a paradox when compared to systemic findings. A prospective study by Guy et al. found that RA and ankylosing spondylitis (AS) patients had lower levels of plasma renin and a correspondingly high aldosterone-to-renin ratio compared to healthy controls [29]. These systemic findings contrast with reports of increased renin and ACE activity within synovial tissue and fluid, suggesting a dissociation between circulating and joint-local RAS activity. Overall, such observations are consistent with the concept of a locally regulated, partially autonomous tissue RAS, indicating that systemic measurements may not fully reflect intra-articular pathophysiology [29].

To distinguish between joint-local and systemic RAS activity, the biological compartments and sample types used for RAS assessment in RA and OA are summarized in Table 3.

**Table 3:** Joint-local versus systemic assessment of renin–angiotensin system components in rheumatoid arthritis and osteoarthritis

RAS assessment level	Biological compartment/ sample	Measured components (examples)	Primary interpretation
Joint-local	Synovial tissue	Renin, ACE, ACE2, AT1R, AT2R, MasR, NEP/CD10	Reflects locally regulated RAS activity within the inflamed joint microenvironment
Joint-local	Synovial fluid	Renin, ACE, Ang II, Ang-(1–7), VEGF, MMP-13	Indicates active intra-articular RAS signaling linked to inflammation and tissue damage
Joint-local	Articular cartilage / chondrocytes	AT1R, AT2R, AGT, MMP-13	May represent RAS-mediated catabolic and hypertrophic processes in cartilage
Systemic	Serum / plasma	Renin, ACE, ACE2, Ang II, aldosterone	Reflects circulating RAS activity and systemic cardiovascular or metabolic regulation
Systemic	Peripheral blood	ACE I/D genotype, circulating cytokines	Indicates genetic susceptibility or systemic inflammatory context rather than joint-specific activity

ACE, angiotensin-converting enzyme; ACE2, angiotensin-converting enzyme 2; AGT, angiotensinogen; Ang II, angiotensin II; AT1R, angiotensin II type 1 receptor; AT2R, angiotensin II type 2 receptor; I/D, insertion/deletion polymorphism; MasR, Mas receptor; MMP-13, matrix metalloproteinase-13 (collagenase-3); NEP, neprilysin (CD10); RAS, renin–angiotensin system; VEGF, vascular endothelial growth factor

### The classical axis as a driver of synovial inflammation and pannus formation

In RA, the hyperactivity of the classical ACE/Ang II/AT1R axis is linked to pathogenic mechanisms and clinical disease activity [4].

Mechanistically, the classical RAS contributes to pathways associated with the development of the aggressive, “tumor-like” synovial pannus, a hallmark of RA. Pattacini et al. demonstrated that Ang II protects RA fibroblast-like synoviocytes (FLS) derived from RA patients—the main cellular component of the pannus—from apoptosis caused by serum starvation or nitric oxide [30]. This potent anti-apoptotic effect was mediated via the AT1R and activates the pro-survival NF-kB pathway [30].

Furthermore, the RAS functions as an upstream driver of the key cytokines that sustain RA. In an adjuvant-induced arthritis (AIA) rat model, treatment with the direct renin inhibitor aliskiren significantly reduced serum levels of the main RA cytokines tumor necrosis factor (TNF)-alpha and interleukin (IL)-6 [31].

Clinically, Tomaz Braz et al. found that plasma Ang II concentrations positively correlate with the Disease Activity Score 28 (DAS28), establishing a link between the primary pathogenic peptide of the RAS and the clinical severity of RA [32].

### RAS-mediated angiogenesis and bone destruction

The destructive power of the RA pannus depends on two processes, both of which are supported by the local RAS: angiogenesis (to supply the pannus) and bone erosion (the result of pannus invasion) [28, 33].

The invasive pannus is highly vascularized, and the local RAS seems to serve as the “supply line.” Wu et al. identified a significant positive correlation between RAS components (renin, ACE) levels and the potent angiogenic factor VEGF in the synovial fluid of RA patients [28]. In arthritic rat models, systemic Ang II was shown to exacerbate vascular damage, while complementary in vitro experiments demonstrated that Ang II promotes endothelial cell migration and tube formation—key steps in angiogenesis—via ATR/ERK1/2 signaling [34].

The local RAS has been implicated as a mediator of RA-associated bone erosion. Wang et al. provided a molecular mechanism for how the synovial RAS contributes to periarticular osteopenia in a collagen-induced arthritis (CIA) rat model [33]. The study indicated that the RAS launches a *dual-pronged attack* on bone homeostasis:

First, RAS activation increases bone resorption by upregulating the receptor activator of nuclear factor κB ligand (RANKL)/receptor activator of nuclear factor κB (RANK)/tumor necrosis factor receptor–associated factor 6 (TRAF6) signaling pathway, resulting in a higher RANKL/osteoprotegerin (OPG) ratio and enhanced osteoclast differentiation [33]. Second, it suppresses bone formation by increasing Dickkopf-related protein 1 (DKK-1), thereby inhibiting Wnt/β-catenin signaling and osteoblast differentiation [33].

This finding suggests a broader role of the RAS beyond inflammation, potentially acting as a regulator of the uncoupling between osteoclasts and osteoblasts, which underpins the irreversible joint destruction and bone erosions characteristic of RA.

### Systemic implications: RAS and cardiovascular risk in RA

The pathogenic effects of RAS dysregulation in RA are not limited to the joint. Tomaz Braz et al. expanded their research to explore the high CV morbidity and mortality observed in RA patients [32]. They found that levels of the protective enzyme ACE2 negatively correlated with the intima-media thickness (IMT) of the common carotid artery, a key marker of subclinical atherosclerosis [32]. This suggests that the systemic RAS imbalance observed in RA patients (e.g., high Ang II and reduced ACE2 activity) correlates with RA disease activity (DAS28). Also it is associated with markers of subclinical atherosclerosis, linking inflammatory burden with CV risk [32]. Together, these findings support the concept that local joint RAS activation and systemic RAS dysregulation may represent interconnected, parallel processes contributing to both articular disease and extra-articular manifestations in RA.

Box 1: Evidence-tier overview for local RAS involvement in RA **In vitro / ex vivo evidence**:

Cell- and tissue-based studies provide mechanistic data on local RAS activity within the joint, including increased FLS synoviocyte survival, resistance to apoptosis, pro-angiogenic signaling, and dysregulation of bone homeostasis pathways.

**Animal model evidence**:

Experimental arthritis models support a pathogenic role of RAS signaling in synovial inflammation, angiogenesis, and periarticular bone remodeling. In these models, pharmacological modulation of RAS components influences inflammatory cytokine production, vascular changes, and osteoclast–osteoblast balance.

**Human evidence**:

Studies analyzing synovial fluid and synovial tissue from patients with RA demonstrate increased local expression and activity of RAS components compared with OA and healthy controls. Observational human data support associations between circulating or local RAS mediators and disease activity and subclinical CV risk.

**Interpretation**:

The available evidence suggests a role of local RAS activation in RA pathogenesis. However, while human data suggest clinical associations and tissue-level relevance, many mechanistic and pathogenic implications are primarily derived from preclinical models and should therefore be regarded as hypothesis-generating.

## The local RAS in OA pathogenesis

While RA is characterized by synovial inflammation, OA is a whole-joint disease mainly defined by cartilage breakdown and abnormal subchondral bone remodeling [3]. The local RAS also appears to contribute to the disease process in the OA context, albeit through distinct cellular targets and mechanisms [3].

### RAS component expression in articular chondrocytes

The main evidence for RAS in OA pathology comes from the cartilage itself. Kawakami et al. demonstrated that human articular chondrocytes (isolated from OA, RA, and trauma patients) express the mRNA for both AT1R and AT2R [35]. This study suggested that chondrocyte as a RAS-responsive cell. Also, the study found that stimulating these chondrocytes with the pro-inflammatory cytokine IL-1beta—a key mediator in OA pathogenesis—significantly increased the expression of both AT1R and AT2R [35]. These findings suggest the presence of a putative feed-forward mechanism, whereby low-grade mechanical or metabolic inflammation (which produces IL-1beta) may sensitize the chondrocytes through upregulating Ang II receptors [35]. This mechanotransducive modulation may help distinguish OA from the immune-dominant microenvironment of RA.

### RAS-driven cartilage degradation and chondrocyte hypertrophy

The local OA RAS has been implicated in processes associated with cartilage degradation. The review by Wu et al. reported that RAS components are involved in OA-related inflammation and pathogenic chondrocyte hypertrophy [36].

While RAS component levels were lower in OA than in RA, they were still elevated compared to healthy controls and, most importantly, were positively correlated with matrix metalloproteinase-13 (MMP-13, also known as collagenase-3) –which is the primary matrix metalloproteinase responsible for degrading type II collagen, the main structural component of articular cartilage- levels in the synovial fluid [28]. Alexander et al. demonstrated elevated serum MMP-13 levels in end-stage arthroplasty patients, underscoring the central role of this enzyme in cartilage destruction [37]. In the context of preclinical data linking local RAS signaling to MMP-13 upregulation, these clinical observations support the concept that RAS activity may contribute to OA progression through downstream matrix-degrading pathways, rather than representing a purely reactive epiphenomenon.

### Crosstalk with inflammatory signaling pathways

The local RAS does not operate in isolation; it interacts with other key inflammatory pathways in the chondrocyte. A study by Wang et al. outlined a detailed molecular mechanism for RAS-driven OA inflammation [38]. Using an in vitro OA model (IL-6-stimulated human chondrocytes), they found that angiotensinogen (AGT), the precursor for all angiotensins, promotes inflammatory responses, including the production of IL-1beta and MMP-13 [38].

The mechanism behind this effect involved the activation of the Janus kinase 2 (JAK2)/ signal transducer and activator of transcription 3 (STAT3) signaling pathway [31, 38, 39]. This activation may result from AGT, since AGT knockdown or a JAK inhibitor attenuated the inflammatory response [31, 38]. This finding is especially relevant when considered alongside the RA data. Azouz et al. showed in an RA model that the renin inhibitor aliskiren blocks the same IL-6/JAK2/STAT3 pathway [31]. These findings suggest that local RAS activity may converge on the IL-6/JAK2/STAT3 signaling pathway in both RA and OA. While direct clinical implications cannot yet be inferred, this convergence highlights a shared inflammatory signaling node that is already therapeutically targetable in rheumatology (e.g. tofacitinib), underscoring the translational relevance of further investigating RAS–JAK/STAT interactions [31, 38].

### The systemic-local link: the “vascular aetiology hypothesis”

Beyond the joint, the RAS provides a mechanistic link between OA and its most common comorbidities, particularly hypertension and obesity [40, 41]. Ching et al. (2021) proposed a “vascular aetiology hypothesis” that reframing OA in a substantial subset of patients as a manifestation of systemic vascular disease rather than a purely mechanical disorder [40].

This hypothesis suggests that hypertension—a typical high-RAS condition— may act as a causal factor for OA through two separate mechanisms:

1. **Biophysical**: Systemic high blood pressure raises intraosseous pressure in the subchondral bone, causing local hypoxia and triggering abnormal, OA-like bone remodeling at the osteochondral junction [40].
2. **Biochemical**: Systemic activation of the RAS and endothelin systems (which cause hypertension) may locally disrupt joint homeostasis by interfering with key signaling pathways, such as the Wnt/beta-catenin pathway [40].

This perspective expands the concept of “metabolic OA” to include a critical vascular component and positions the RAS as a plausible biological bridge between systemic vascular pathology and local joint degeneration [40, 42].

Box 2. Evidence-tier overview for local RAS involvement in OA **In vitro / ex vivo evidence**:

Cell- and tissue-based studies provide mechanistic data on local RAS activity within the joint, including increased FLS synoviocyte survival, resistance to apoptosis, pro-angiogenic signaling, and dysregulation of bone homeostasis pathways.

**Animal model evidence**:

Experimental models support RAS-mediated cartilage degeneration, subchondral bone remodeling, and vascular mechanisms contributing to OA-like changes.

**Human evidence**:

Human studies report expression of RAS receptors in articular chondrocytes and associations between local RAS components and markers involved in cartilage degradation, such as MMP-13. Observational studies have also reported links between systemic RAS activation, vascular comorbidities, and OA phenotypes.

**Interpretation**:

Available evidence supports a role for local and systemic RAS signaling in OA pathogenesis. However, most mechanistic and therapeutic implications are derived from preclinical and in vitro studies and should therefore be regarded as hypothesis-generating.

## Genetic predisposition: the ACE insertion/deletion (I/D) polymorphism

Given the pathogenic role of the classical ACE/Ang II/AT1R axis, genetic differences in the ACE gene have been a focus of research. The ACE I/D polymorphism, in which the ‘D’ (deletion) allele is linked to higher levels of circulating and tissue ACE, has been studied in both RA and OA, revealing a disease-selective and heterogeneous pattern of associations.

### Association with RA susceptibility

Several studies suggest a more consistent association between the ‘D’ allele, particularly the homozygous ‘DD’ genotype, and susceptibility to RA. In an Egyptian cohort, Ahmed et al. found that the ‘DD’ genotype was associated with a 5.6-fold increased risk of RA. ‘D’ allele carriers generally had a 2.8-fold increased risk [43]. Uppal et al. found a significant overrepresentation of the ‘DD’ genotype and the ‘D’ allele in RA patients, calculating a 3.0-fold increase in relative risk for the ‘DD’ genotype [44]. At the meta-analysis level, Song et al., including five RA studies, reported a pooled association between the ACE I/D polymorphism and RA susceptibility (Odds Ratio (OR) 2.199 for the DD + ID genotype) [45], with a stronger signal observed in Arab populations (OR 2.697 for the D allele), consistent with the results from the Egyptian and Kuwaiti cohorts [45]. However, the authors also noted between-study heterogeneity, underscoring the population-dependent nature of this association.

A key nuance, however, is that this genetic association seems to be limited to susceptibility rather than severity. Ahmed et al. found limited and statistically non-significant association between the ‘DD’ genotype and clinical disease activity (DAS28) or radiological damage (Sharp’s score) in established RA patients [43].

This association is not universal, due to the population-specific nature of genetic associations [46]. Ghelani et al., in an analysis of RA-related genes in UK South Asian and Caucasian populations, did not report a significant link for ACE, but instead found connections for tumor necrosis factor receptor 2 (TNFR2) and vitamin D receptor (VDR), emphasizing the impact of ethnicity on genetic risk factors [47].

### Association with OA susceptibility

In contrast to the RA data, studies examining the ACE I/D polymorphism in OA have reported generally weak, inconsistent, or non-significant associations. Lin et al. conducted a case-control study and a meta-analysis (totaling 1165 cases and 1029 controls) specifically examining the connection between this polymorphism and knee OA [48]. Notably, although the pooled analysis did not reach statistical significance, the meta-analysis showed a numerical trend toward increased risk (OR 1.37) accompanied by very high between-study heterogeneity (I² = 87.2%). This degree of heterogeneity suggests a complex, multifactorial genetic architecture and raises the possibility that context-specific effects or gene–gene interactions may modulate any potential association between ACE I/D and knee OA [48].

A summary of key population-based studies and meta-analyses examining the association between the ACE I/D polymorphism and arthritis susceptibility, highlighting differences between RA and OA and population-specific effects, is provided in Table 4.

**Table 4:** Summary of studies on ACE I/D polymorphism and arthritis susceptibility

Population/study type	Disease	Key finding	References
Egyptian	RA	Positive association with RA susceptibility. 5.6-fold risk for ‘DD’ genotype. Limited and statistically non-significant association with RA severity (DAS28, Sharp’s).	[43]
Kuwait	RA	Positive association with RA susceptibility. 3.0-fold relative risk for ‘DD’ genotype.	[44]
Meta-analysis (5 studies)	RA	Positive association with RA susceptibility (OR 2.199). Association particularly strong in Arab populations (OR 2.697).	[45]
UK South Asian & Caucasian	RA	Limited but statistically non-significant association with ACE I/D in this population, highlighting population-specific and ethnicity-dependent genetic effects	[47]
Case-control & Meta-analysis	Knee OA	Limited and inconsistent evidence for an association with knee OA susceptibility, with high between-study heterogeneity (allele model OR 1.37 [0.95–1.99])	[48]

ACE: Angiotensin converting enzyme, DAS: Disease Activity Score, OA: Osteoarthritis, OR: Odds ratio, RA: Rheumatoid arthritis, D: Deletion

This apparent genetic divergence may be biologically relevant. Increased ACE and Ang II levels associated with the ‘D’ allele may contribute more consistently to immune-driven, synovial-dominant processes characteristic of RA. In contrast, available evidence does not support a consistent association with predominantly mechanical or metabolic, cartilage-based pathology of OA [48]. These observations suggest that ACE I/D–related effects are more likely linked to immune–inflammatory mechanisms of the classical RAS axis—such as FLS survival, immune cell recruitment, and synovial angiogenesis—rather than to cartilage metabolism alone, processes that are central in RA but appear secondary in OA [3, 4].

## Therapeutic targeting of the local joint RAS

The preclinical studies suggest that targeting RAS may be therapeutical relevance in both RA and OA [49, 50]. On this basis, two broad approaches have been explored: (i) evaluating conventional RAS inhibitors in experimental arthritis/OA models and observational human studies, and (ii) developing strategies that enhance the protective ACE2/Ang-(1–7) axis.

### Conventional antihypertensives (classical axis blockade)

Most evidence supporting the effects of conventional antihypertensive agents on joint-local RAS signalling derives from preclinical arthritis/OA models; human data are limited and largely observational. The anti-inflammatory effects of conventional antihypertensives, such as angiotensin-converting enzyme inhibitors (ACEi) and angiotensin receptor blockers (ARBs), have been studied in various inflammatory conditions, including arthritis [31, 51, 52].

#### In OA models

Preclinical data support a chondroprotective role for these agents. Tang et al. showed that the ACEi captopril had chondroprotective effects in a rat model of OA [51]. Captopril treatment slowed cartilage degeneration, limited the abnormal expansion of the hypertrophic zone of chondrocytes, and was associated with the suppression of the local joint RAS components [51]. Likewise, Yan et al. demonstrated that the direct renin inhibitor aliskiren also offered chondroprotective effects in a rat OA model, attenuating cartilage damage and hypertrophic chondrocyte expansion, accompanied by suppression of the local RAS components [52].

#### In RA models

Azouz et al. tested aliskiren (a renin inhibitor) in an AIA rat model and found its efficacy was reported by authors as “close to those of.methotrexate,” the gold-standard disease-modifying antirheumatic drug (DMARD) for RA, however, human data is needed [31]. Aliskiren potently reduced TNF-alpha, IL-6, and the key biomarkers of joint destruction, MMP-3 and RANKL [31]. As noted, this effect was linked to the downregulation of the IL-6/JAK2/STAT3 pathway [31]. Wang et al. showed that the AT1R blocker losartan reduced arthritis in AIA rats [53].

These preclinical studies suggest that classical-axis blockade can reduce inflammatory readouts and structural damage markers, including cartilage degradation and bone remodeling. However, whether these effects translate into clinically meaningful outcomes in humans remains uncertain. For clarity, the main experimental therapeutic strategies targeting the joint RAS, along with their preclinical models and reported molecular or pathological outcomes, are summarized in Table 5.

**Table 5:** Overview of therapeutic strategies targeting the joint Renin angiotensin system

Class	Agent(s)	Model	Key Molecular / Pathological Outcomes	Refs.
*Pathogenic Axis Inhibition*				
Renin Inhibitor	Aliskiren	Rat OA Model	$\:\downarrow\:\:$RAS components, Chondroprotective	[52]
	Aliskiren	Rat AIA (RA) Model	↓TNF-alpha, ↓IL-6, ↓MMP-3, ↓RANKL, ↓p-JAK2/p-STAT3. Reported preclinical efficacy “close to methotrexate”, but human data needed.	[31]
ACE Inhibitor	Captopril	Rat OA Model	↓ Local RAS, Attenuated cartilage degeneration, Partially reversed chondrocyte abnormalities	[51]
AT1R Blocker (ARB)	Losartan	Rat AIA (RA) Model	Ameliorated arthritis, ↑AT2R expression (compensatory upregulation)	[53]
*Protective Axis Activation*				
ACE2 Activator	Diminazene Aceturate (DIZE)	Rodent OA Model	↑ Ang-(1–7), ↓Inflammation & oxidative stress. ‘’*More prominent effect than losartan*.’’ in this preclinical model	[54]
AT2R Agonist	CGP42112	Rat AIA (RA) Model	Ameliorated arthritis index & histology	[53]
	Novokinin	Rat AIA (RA) Model	Restored RAS balance, ↓Inflammatory (HETEs) & ↑Anti-inflammatory (EETs) metabolites	[55]
*Advanced Delivery*				
Bone-Targeting Conjugate	Ang-(1–7)-Bisphosphonate	Rat AIA (RA) Model	Extended half-life, Enhanced anti-inflammatory effect, ↓ Joint edema & serum NO	[56]
Bone-Targeting Conjugate	Novokinin-Bisphosphonate	Rat AIA (RA) Model	Improved stability, Enhanced anti-inflammatory effects	[55]

ACE: Angiotensin-Converting Enzyme, ACE2: Angiotensin-Converting Enzyme 2, AIA: Adjuvant-Induced Arthritis, Ang-(1–7): Angiotensin 1–7 peptide, ARB: Angiotensin II Receptor Blocker, AT1R: Angiotensin II Type 1 Receptor, AT2R: Angiotensin II Type 2 Receptor, CGP42112: Selective AT2R agonist compound, DIZE: Diminazene Aceturate, EETs: Epoxyeicosatrienoic Acids, HETEs: Hydroxyeicosatetraenoic Acids, IL-6: Interleukin-6, JAK2: Janus Kinase 2, MMP-3: Matrix Metalloproteinase-3, NO: Nitric Oxide, OA: Osteoarthritis, p-STAT3: Phosphorylated Signal Transducer and Activator of Transcription 3, RAS: Renin–Angiotensin System, RA: Rheumatoid Arthritis, RANKL: Receptor Activator of Nuclear Factor-κB Ligand, TNF-α: Tumor Necrosis Factor-alpha

### Activating the protective axis

Another approach involves not just blocking the harmful axis but also activating the protective ACE2/Ang-(1–7)/MasR axis.

Habib et al. conducted a direct comparison in a rodent OA model, testing losartan (AT1R blockade) against diminazene aceturate (DIZE), a known activator of the protective enzyme ACE2 in preclinical studies, although its precise mechanism and translational potential remain debated [54]. While both drugs improved OA findings, DIZE showed a larger effect on inflammatory and oxidative stress readouts in that model compared to losartan, highlighting the potential benefit of targeting the protective pathway in experimental settings.

The protective AT2R is also another target. Wang et al. reported that treatment in AIA rats not only blocked AT1R but also increased AT2R expression [53]. They then demonstrated that a direct AT2R agonist (CGP42112) also reduced findings of arthritis [53]. Ranjit et al. tested a synthetic AT2R agonist, novokinin, in an AIA model, finding that it restored RAS balance and produced anti-inflammatory effects by shifting the arachidonic acid pathway from inflammatory metabolites (hydroxyeicosatetraenoic acids [HETEs]) toward protective ones (epoxyeicosatrienoic acids [EETs]) [55].

### Advanced delivery for enhanced efficacy: bone-targeting conjugates

A major challenge in using protective peptides like Ang-(1–7) or novokinin as medications is their extremely short plasma half-life, which limits their therapeutic potential [55]. To address this, advanced drug-delivery systems have been developed.

Pour et al. (for Ang-(1–7)) and Ranjit et al. (for novokinin) engineered new conjugates by chemically attaching these protective peptides to a bisphosphonate moiety [55, 56]. Bisphosphonates strongly bind to hydroxyapatite, the mineral in bone. This design enables the Ang-(1–7) conjugate (“Ang. Conj.“) or novokinin conjugate (“Novo Conj.“) to target the bone and joints, effectively using the bone as a local reservoir for the sustained release of the protective peptide at the site of inflammation [55, 56]. This approach may protect the peptide from systemic breakdown, prolongs its half-life, and was associated with stronger and more sustained anti-inflammatory effects in arthritis models [55, 56].

### Interaction with current rheumatologic therapies (DMARDs)

A critical and complex question is how these local RAS pathways interact with our *current* RA drugs.

Soos et al. studied RA and AS patients undergoing one year of anti-TNF therapy [57]. They found that treatment increased serum ACE levels but also increased the activity of the protective enzyme ACE2. This suggests that anti-TNF therapy may induce a complex yet potentially relevant modulation of the RAS [57].

Conversely, findings related to JAK inhibition raise important questions. Kacsandi et al. studied RA patients on tofacitinib (a JAK inhibitor) for one year [58]. They found that tofacitinib increased serum ACE levels but also increased the ACE/ACE2 ratio [58]. An increased ACE/ACE2 ratio may indicate a shift away from the protective axis and toward the pathogenic classical axis. The authors stated that: “The effect of tofacitinib on RAS may provide a plausible explanation for the CV effects of JAK inhibition in RA.“; which may offer one possible mechanistic hypothesis among several proposed explanations for CV risk signals observed with JAK inhibition [58]. This complicates treatment strategies and signals a move toward personalized medicine, where the selection of DMARDs and the potential addition of RAS modulators must be carefully evaluated considering their complex interaction and the patient’s CV risk profile [58]. The observed association between DMARD use, particularly JAK inhibition, and changes in the ACE/ACE2 balance should be interpreted with caution. Available evidence in this area is derived mainly from observational analyses and post hoc safety evaluations and therefore does not allow causal inference. In this context, the proposed link between JAK inhibitors, local RAS modulation, and CV outcomes should be regarded as a plausible hypothesis rather than a demonstrated mechanism. Concerns regarding CV risk with JAK inhibitors have been raised by large safety studies, most notably the ORAL Surveillance trial, which reported higher rates of major adverse CV events with tofacitinib compared with TNF inhibitors in patients with RA and increased baseline CV risk [59]. However, several alternative and potentially overlapping explanations may contribute to these observations, including differences in underlying inflammatory burden, serologic status, baseline CV comorbidities, concomitant therapies, and broader effects on endothelial function and vascular homeostasis, and suboptimal adherence to cardiovascular risk assessment and management guidelines in routine clinical practice [60–63]. In addition, JAK–STAT signaling intersects with multiple cytokine pathways involved in endothelial activation and metabolic regulation, which may influence CV risk, independent of local RAS modulation, as suggested by recent in vivo studies showing preserved coronary microvascular perfusion during JAK inhibitor therapy in patients with RA [64, 65]. At present, these observations should be interpreted as hypothesis-generating and do not support changes in clinical practice.

To improve transparency and align key claims with their underlying levels of evidence and limitations, the main mechanistic and therapeutic inferences discussed throughout the manuscript are summarized in Table 6.

**Table 6:** Summary of key claims, evidence levels, and limitations regarding joint-local RAS in RA and OA, with emphasis on evidence hierarchy

Claim	Evidence type	Example studies	Comment/limitation
*Rheumatoid Arthritis*			
Local synovial RAS is compartmentalized and upregulated in RA	Human (synovial fluid & tissue)	Çobankara et al.; Wu et al.	Primarily observational; limited causal inference
Classical RAS activation supports synovial hyperplasia and FLS survival	In vitro / ex vivo	Pattacini et al.	Mechanistic data; limited direct validation in human tissue
RAS signaling promotes angiogenesis in RA synovium	Human (correlative) + animal models	Wu et al.; experimental arthritis models	Angiogenic effects largely supported by preclinical studies
Local RAS contributes to bone erosion via RANKL/OPG and Wnt pathways	Animal models	Wang et al. (CIA model)	Disease-modifying implications remain preclinical
Systemic RAS imbalance is associated with increased CV risk in RA	Human observational	Tomaz Braz et al.	Association does not establish causality
*Osteoarthritis*			
Chondrocytes express functional RAS receptors in OA	Human (ex vivo)	Kawakami et al.	Primarily descriptive
Local RAS activity is associated with cartilage degradation	Human (correlative)	Wu et al.; Alexander et al.	Association, not causation
RAS promotes inflammatory and catabolic signaling in chondrocytes	In vitro	Wang et al.	Cell-based models
RAS interacts with IL-6/JAK2/STAT3 signaling in OA	In vitro + preclinical	Wang et al.; Azouz et al.	Limited direct human validation
Systemic RAS activation links OA with vascular comorbidity	Human observational	Ching et al.	Hypothesis-based framework
*Therapeutic Targeting*			
ACEi/ARB/renin inhibition reduces OA-like cartilage damage	Animal models	Tang et al.; Yan et al.	Preclinical endpoints; translation uncertain
Renin inhibition reduces inflammatory cytokines and damage markers in arthritis	Animal models	Azouz et al. (AIA rats)	Model-dependent; not human efficacy
AT1R blockade / AT2R agonism reduces arthritis severity	Animal models	Wang et al.; Ranjit et al.	Preclinical; dosing/target engagement unclear in humans
Bone-targeted peptide conjugates improve arthritis readouts	Animal models	Pour et al.; Ranjit et al.	Delivery concept; human feasibility unknown
DMARDs may modulate systemic RAS biomarkers	Human observational	Soos et al.; Kacsandi et al.	Association only; confounding likely
CV risk signals with tofacitinib require multifactorial interpretation	Human RCT safety signal + observational	ORAL Surveillance; other studies	Not specific to RAS; mechanism unproven

ACE, angiotensin-converting enzyme; ACEi, angiotensin-converting enzyme inhibitor; AIA, adjuvant-induced arthritis; Ang, angiotensin; ARB, angiotensin receptor blocker; AT1R, angiotensin II type 1 receptor; AT2R, angiotensin II type 2 receptor; CIA, collagen-induced arthritis; CV, cardiovascular; DMARDs, disease-modifying antirheumatic drugs; FLS, fibroblast-like synoviocytes; IL, interleukin; JAK, Janus kinase; OA, osteoarthritis; OPG, osteoprotegerin; RA, rheumatoid arthritis; RAS, renin–angiotensin system; RANKL, receptor activator of nuclear factor κB ligand; RCT, randomized controlled trial; STAT, signal transducer and activator of transcription; TNF, tumor necrosis factor; Wnt, Wingless-related integration site

Box 3. Evidence-tier overview for therapeutic targeting of the joint-local RAS **In vitro/ex vivo evidence**:

Cellular studies suggest anti-inflammatory and anti-catabolic effects of RAS modulation (e.g., effects on cytokine production, MMPs, and signaling pathways).

**Animal model evidence**:

Most therapeutic signals—classical-axis blockade (ACEi/ARBs/renin inhibition), protective-axis activation (ACE2/AT2R agonism), and bone-targeted peptide conjugates—are supported primarily by experimental arthritis and OA models with limited cross-validation across species or disease stages.

**Human evidence**:

Human data are currently limited and consist mainly of observational or small mechanistic studies evaluating circulating RAS components in treated patients, alongside CV outcome signals from large safety trials (e.g., tofacitinib vs. TNF inhibitors). Direct evidence that RAS modulation improves clinical or tissue-level joint outcomes in RA/OA patients remains limited.

**Interpretation**:

At present, these therapeutic approaches are best viewed as hypothesis-generating, with preclinical efficacy but limited direct clinical evidence. Future studies should prioritize prospective human investigations, validated biomarkers, and tissue-specific assessments.

## Conclusion and future directions

This review summarizes current evidence indicating that the local renin-angiotensin system may function as an active and locally regulated network within the joint microenvironment, rather than a passive or incidental system. In RA, multiple experimental and translational studies indicate that increased activity of the classical ACE/Ang II/AT1R axis in synovial tissue is associated with key pathological features, including FLS survival through NF-kB–related pathways [30], angiogenesis mediated by VEGF [53], and structural joint damage linked to imbalance between the RANKL and Wnt/beta-catenin pathways [33]. In OA, available data suggest that RAS activity in chondrocytes may contribute to cartilage degradation, for example through MMP-13 induction [28] and to inflammatory signaling involving the JAK2/STAT3 pathway [38].

This review also summarizes disease-selective genetic patterns. The ACE I/D polymorphism has been reported more consistently in association with RA susceptibility, particularly in certain populations, whereas evidence for an association with knee OA is limited or inconsistent, with high between-study heterogeneity [45]. These findings support that genetic links to RAS may be more relevant to immune-driven synovial processes than to predominantly mechanical or metabolic cartilage pathology.

From a therapeutic standpoint, modulation of the local RAS remains an area of interest, but current evidence is mainly preclinical [49]. Experimental studies suggest that RAS inhibitors (ACE inhibitors, ARBs, renin inhibitors), as well as agents targeting the protective axis such as DIZE or Novokinin, may influence inflammatory and structural pathways within the joint in animal models [54]. Whether these effects translate into clinically meaningful benefits in human disease remains to be determined.

However, important questions still exist to define priorities for future research:

1. Translational Studies: A major unmet need is for well-designed prospective studies and randomized clinical trials to assess whether preclinical observations related to local RAS modulation translate into meaningful outcomes in human disease. In particular, it remains unknown whether ARBs, ACE inhibitors, or renin inhibitors can influence structural progression or long-term outcomes in RA or OA.
2. DMARD-RAS Interactions: Observations suggesting that tofacitinib may alter the ACE/ACE2 balance warrant further investigation in carefully controlled studies [58]. Future work may address whether such changes are independently linked to CV risk, regardless of disease activity and baseline comorbidities, and whether concomitant RAS-modulating therapies modify this risk.
3. Biomarkers: The potential value of RAS components or related peptidases measured in synovial fluid as predictive or prognostic biomarkers of disease progression or treatment response remains uncertain [66].
4. The Viral-Rheumatic Link: The detection of Severe Acute Respiratory Syndrome Coronavirus 2 (SARS-CoV-2)in the synovial fluid of a patient with acute arthritis raises questions about whether viral infections may disrupt local ACE2-dependent protective pathways within the joint [67]. Further studies are required to clarify whether viral triggers can influence joint-specific RAS balance and inflammatory responses.
5. Refining Therapeutics: Continued development of bone- and joint-targeted delivery systems may offer an opportunity to study localized modulation of RAS pathways while minimizing systemic exposure. Such approaches may also provide useful experimental tools to understand better the contribution of local RAS activity to articular disease [50].

## Data Availability

All data discussed in the manuscript arederived from previously published literature; therefore, no new datasetswere generated or analyzed.

## References

[1] Triebel H, Castrop H (2024) The Renin angiotensin aldosterone system. Pflugers Arch 476:705–713. 10.1007/s00424-024-02908-1 38233636 10.1007/s00424-024-02908-1 PMC11033231

[2] Paul M, Poyan Mehr A, Kreutz R (2006) Physiology of local renin-angiotensin systems. Physiol Rev 86:747–803. 10.1152/physrev.00036.2005 16816138 10.1152/physrev.00036.2005

[3] Zhao J, Yang H, Chen B, Zhang R (2019) The skeletal renin-angiotensin system: A potential therapeutic target for the treatment of osteoarticular diseases. Int Immunopharmacol 72:258–263. 10.1016/j.intimp.2019.04.023 31003003 10.1016/j.intimp.2019.04.023

[4] Chaves Moreira FR, de Oliveira TA, Ramos NE, Duarte Abreu MA, Simoes E, Silva AC (2021) The role of Renin angiotensin system in the pathophysiology of rheumatoid arthritis. Mol Biol Rep 48:6619–6629. 10.1007/s11033-021-06672-8 34417705 10.1007/s11033-021-06672-8

[5] Paz Ocaranza M, Riquelme JA, García L, Jalil JE, Chiong M et al (2020) Counter-regulatory renin-angiotensin system in cardiovascular disease. Nat Rev Cardiol 17:116–129. 10.1038/s41569-019-0244-8 31427727 10.1038/s41569-019-0244-8 PMC7097090

[6] Bader M, Steckelings UM, Alenina N, Santos RAS, Ferrario CM (2024) Alternative renin-angiotensin system. Hypertension 81:964–976. 10.1161/HYPERTENSIONAHA.123.21364 38362781 10.1161/HYPERTENSIONAHA.123.21364 PMC11023806

[7] Habib YH, Khattab M, Gowayed MA (2025) The renin–angiotensin system (RAS) and arthritic diseases: therapeutic potential for RAS inhibitors. Inflammopharmacology 33:5037–5047. 10.1007/s10787-025-01890-z 40796991 10.1007/s10787-025-01890-z

[8] Qian Y, Zhang B, Nian F (2025) The association between rheumatoid arthritis and left ventricular diastolic dysfunction: pathogenesis, predictors and managements. Clin Exp Rheumatol 43:135–144. 10.55563/clinexprheumatol/kmmkj7 39212131 10.55563/clinexprheumatol/kmmkj7

[9] Gasparyan AY, Ayvazyan L, Blackmore H, Kitas GD (2011) Writing a narrative biomedical review: considerations for authors, peer reviewers, and editors. Rheumatol Int 31:1409–1417. 10.1007/s00296-011-1999-3 21800117 10.1007/s00296-011-1999-3

[10] Almutlaq M, Alamro AA, Alroqi F, Barhoumi T (2021) Classical and counter-regulatory renin–angiotensin system: potential key roles in COVID-19 pathophysiology. CJC Open 3:1060–1074. 10.1016/j.cjco.2021.04.004 33875979 10.1016/j.cjco.2021.04.004 PMC8046706

[11] Kanugula AK, Kaur J, Batra J, Ankireddypalli AR, Velagapudi R (2023) Renin-angiotensin system: updated Understanding and role in physiological and pathophysiological States. Cureus 15:e40725. 10.7759/cureus.40725 37350982 10.7759/cureus.40725 PMC10283427

[12] Pejler G, Rönnberg E, Waern I, Wernersson S (2010) Mast cell proteases: multifaceted regulators of inflammatory disease. Blood 115:4981–4990. 10.1182/blood-2010-01-257287 20233968 10.1182/blood-2010-01-257287

[13] Nigrovic PA, Lee DM (2005) Mast cells in inflammatory arthritis. Arthritis Res Ther 7:1–11. 10.1186/ar1446 15642148 10.1186/ar1446 PMC1064877

[14] Loucks A, Maerz T, Hankenson K, Moeser A, Colbath A (2023) The multifaceted role of mast cells in joint inflammation and arthritis. Osteoarthritis Cartilage 31:567–575. 10.1016/j.joca.2023.01.005 36682447 10.1016/j.joca.2023.01.005

[15] Chang Y, Wei W (2015) Angiotensin II in inflammation, immunity and rheumatoid arthritis. Clin Exp Immunol 179:137–145. 10.1111/cei.12467 25302847 10.1111/cei.12467 PMC4298392

[16] Nguyen G, Delarue F, Burcklé C, Bouzhir L, Giller T et al (2002) Pivotal role of the Renin/prorenin receptor in angiotensin II production and cellular responses to Renin. J Clin Invest 109:1417–1427. 10.1172/jci14276 12045255 10.1172/JCI14276 PMC150992

[17] Wang B, Jie H, Wang S, Dong B, Zou Y (2023) The role of (pro)renin receptor and its soluble form in cardiovascular diseases. Front Cardiovasc Med 10:1086603. 10.3389/fcvm.2023.1086603 36824459 10.3389/fcvm.2023.1086603 PMC9941963

[18] Haque R, Iuvone PM, He L, Choi KSC, Ngo A et al (2017) The MicroRNA-21 signaling pathway is involved in prorenin receptor (PRR)-induced VEGF expression in ARPE-19 cells under a hyperglycemic condition. Mol Vis 23:251–262 PMID:28465657 28465657 PMC5398881

[19] Domenig O, Manzel A, Grobe N, Königshausen E, Kaltenecker CC et al (2016) Neprilysin is a mediator of alternative renin-angiotensin-system activation in the murine and human kidney. Sci Rep 6:33678. 10.1038/srep33678 27649628 10.1038/srep33678 PMC5030486

[20] Matucci-Cerinic M, Lombardi A, Leoncini G, Pignone A, Sacerdoti L et al (1993) Neutral endopeptidase (3.4.24.11) in plasma and synovial fluid of patients with rheumatoid arthritis. A marker of disease activity or a regulator of pain and inflammation? Rheumatol Int 13:1–4. 10.1007/BF00290326 8390712 10.1007/BF00290326

[21] Lautner RQ, Villela DC, Fraga-Silva RA, Silva N, Verano-Braga T et al (2013) Discovery and characterization of alamandine: a novel component of the renin-angiotensin system. Circ Res 112:1104–1111. 10.1161/circresaha.113.301077 23446738 10.1161/CIRCRESAHA.113.301077

[22] Rukavina Mikusic NL, Silva MG, Erra Díaz FA, Pineda AM, Ferragut F et al (2024) Alamandine, a protective component of the renin-angiotensin system, reduces cellular proliferation and interleukin-6 secretion in human macrophages through MasR–MrgDR heteromerization. Biochem Pharmacol 229:116480. 10.1016/j.bcp.2024.116480 39128587 10.1016/j.bcp.2024.116480

[23] de Carvalho Santuchi M, Dutra MF, Vago JP, Lima KM, Galvão I et al (2019) Angiotensin-(1–7) and alamandine promote anti-inflammatory response in macrophages in vitro and in vivo. Mediators Inflamm 2019:2401081. 10.1155/2019/2401081 10.1155/2019/2401081 PMC6409041 30918468

[24] Wang Y, Del Borgo M, Lee HW, Baraldi D, Hirmiz B et al (2017) Anti-fibrotic potential of AT(2) receptor agonists. Front Pharmacol 8:564. 10.3389/fphar.2017.00564 28912715 10.3389/fphar.2017.00564 PMC5583590

[25] Abadir PM, Foster DB, Crow M, Cooke CA, Rucker JJ et al (2011) Identification and characterization of a functional mitochondrial angiotensin system. Proc Natl Acad Sci U S A 108:14849–14854. 10.1073/pnas.1101507108 21852574 10.1073/pnas.1101507108 PMC3169127

[26] Berry C, Touyz R, Dominiczak AF, Webb RC, Johns DG (2001) Angiotensin receptors: signaling, vascular pathophysiology, and interactions with ceramide. Am J Physiol Heart Circ Physiol 281:H2337–H2365. 10.1152/ajpheart.2001.281.6.H2337 11709400 10.1152/ajpheart.2001.281.6.H2337

[27] Çobankara V, Öztürk MA, Kiraz S, Ertenli I, Haznedaroglu I et al (2005) Renin and angiotensin-converting enzyme (ACE) as active components of the local synovial renin-angiotensin system in rheumatoid arthritis. Rheumatol Int 25:285–291. 10.1007/s00296-004-0564-8 15761728 10.1007/s00296-004-0564-8

[28] Wu Y, Li M, Zeng J, Feng Z, Yang J et al (2020) Differential expression of renin-angiotensin system-related components in patients with rheumatoid arthritis and osteoarthritis. Am J Med Sci 359:17–26. 10.1016/j.amjms.2019.10.014 31785770 10.1016/j.amjms.2019.10.014

[29] Guy A, Sharif K, Bragazzi NL, Krosser A, Gilburd B et al (2018) Low levels of Renin and high aldosterone-to-renin ratio among rheumatoid patients and ankylosing spondylitis patients: A prospective study. Isr Med Assoc J 20:632–636 PMID:30324781 30324781

[30] Pattacini L, Casali B, Boiardi L, Pipitone N, Albertazzi L et al (2007) Angiotensin II protects fibroblast-like synoviocytes from apoptosis via the AT1-NF-κB pathway. Rheumatology (Oxford) 46:1252–1257. 10.1093/rheumatology/kem092 17526929 10.1093/rheumatology/kem092

[31] Azouz AA, Saleh E, Abo-Saif AA (2020) Aliskiren, tadalafil, and cinnamaldehyde alleviate joint destruction biomarkers; MMP-3 and RANKL; in complete freund’s adjuvant arthritis model: downregulation of IL-6/JAK2/STAT3 signaling pathway. Saudi Pharm J 28:1101–1111. 10.1016/j.jsps.2020.07.011 32922141 10.1016/j.jsps.2020.07.011 PMC7474170

[32] Tomaz Braz NF, Pinto MRC, Marciano Vieira EL, Souza AJ, Teixeira AL et al (2021) Renin-angiotensin system molecules are associated with subclinical atherosclerosis and disease activity in rheumatoid arthritis. Mod Rheumatol 31:119–126. 10.1080/14397595.2020.1740418 32149558 10.1080/14397595.2020.1740418

[33] Wang Y, Kou J, Zhang H, Wang C, Li H et al (2018) The renin-angiotensin system in the synovium promotes periarticular osteopenia in a rat model of collagen-induced arthritis. Int Immunopharmacol 65:550–558. 10.1016/j.intimp.2018.11.001 30412852 10.1016/j.intimp.2018.11.001

[34] Zhang Y, Luo X, Zhou Y, Wu H, Chen J et al (2017) 2K1C-activated angiotensin II (Ang II) exacerbates vascular damage in a rat model of arthritis through the ATR/ERK1/2 signaling pathway. Inflamm Res 66:881–890. 10.1007/s00011-017-1069-8 28653218 10.1007/s00011-017-1069-8

[35] Kawakami Y, Matsuo K, Murata M, Yudoh K, Nakamura H et al (2012) Expression of angiotensin II receptor-1 in human articular chondrocytes. Arthritis 2012:648537. 10.1155/2012/648537 23346400 10.1155/2012/648537 PMC3546464

[36] Wu Y, Lu X, Li M, Zeng J, Zeng J et al (2019) Renin-angiotensin system in osteoarthritis: A new potential therapy. Int Immunopharmacol 75:105796. 10.1016/j.intimp.2019.105796 31408841 10.1016/j.intimp.2019.105796

[37] Alexander K, Banos A, Abro S, Hoppensteadt D, Fareed J et al (2016) Levels of matrix metalloproteinases in arthroplasty patients and their correlation with inflammatory and thrombotic activation processes. Clin Appl Thromb Hemost 22:441–446. 10.1177/1076029616639704 27052781 10.1177/1076029616639704

[38] Wang W, Han X, Zhao T, Zhang X, Qu P et al (2020) AGT, targeted by miR-149-5p, promotes IL-6-induced inflammatory responses of chondrocytes in osteoarthritis via activating the JAK2/STAT3 pathway. Clin Exp Rheumatol 38:1088–1095 PMID:32141427 32141427

[39] Haznedaroglu IC, Arici M, Büyükaşik Y (2000) A unifying hypothesis for the renin-angiotensin system and hematopoiesis: sticking the pieces together with the JAK-STAT pathway. Med Hypotheses 54:80–83. 10.1054/mehy.1998.0830 10790731 10.1054/mehy.1998.0830

[40] Ching K, Houard X, Berenbaum F, Wen C (2021) Hypertension Meets osteoarthritis - revisiting the vascular aetiology hypothesis. Nat Rev Rheumatol 17:533–549. 10.1038/s41584-021-00650-x 34316066 10.1038/s41584-021-00650-x

[41] Pimenta S, Gonçalves H, Pimenta M, Martins A, Costa L et al (2025) Central obesity and radiographic severity are associated with symptomatic hand osteoarthritis: a population-based cross-sectional study. Rheumatol Int 45:143–155. 10.1007/s00296-025-05891-7 40382498 10.1007/s00296-025-05891-7 PMC12085380

[42] Luisa Bonet M, Granados N, Palou A (2011) Molecular players at the intersection of obesity and osteoarthritis. Curr Drug Targets 12:2103–2128. 10.2174/138945011798829393 22023406 10.2174/138945011798829393

[43] Ahmed AZ, El-Shahaly HA, Omar AS, Ghattas MH (2013) Patterns of angiotensin converting enzyme insertion/deletion gene polymorphism among an Egyptian cohort of patients with rheumatoid arthritis. Int J Rheum Dis 16:284–290. 10.1111/j.1756-185X.2012.01820.x 23981749 10.1111/j.1756-185X.2012.01820.x

[44] Uppal SS, Haider MZ, Hayat SJ, Abraham M, Sukumaran J et al (2007) Significant association of insertion/deletion polymorphism of the angiotensin-converting enzyme gene with rheumatoid arthritis. J Rheumatol 34:2395–2399 PMID:17985406 17985406

[45] Song GG, Bae S-C, Kim J-H, Lee YH (2015) The angiotensin-converting enzyme insertion/deletion polymorphism and susceptibility to rheumatoid arthritis, vitiligo and psoriasis: A meta-analysis. J Renin Angiotensin Aldosterone Syst 16:195–202. 10.1177/1470320313478285 23413281 10.1177/1470320313478285

[46] Xiong Q, Zhu J, Hasty KA, Canale ST, Stuart J et al (2008) The complexity in hunting for candidate genes within QTL that determine susceptibility to arthritis in rats. Crit Rev Immunol 28:127–157. 10.1615/critrevimmunol.v28.i2.30 18540828 10.1615/critrevimmunol.v28.i2.30

[47] Ghelani AM, Samanta A, Jones AC, Mastana SS (2011) Association analysis of TNFR2, VDR, A2M, GSTT1, GSTM1, and ACE genes with rheumatoid arthritis in South Asians and Caucasians of East Midlands in the United Kingdom. Rheumatol Int 31:1355–1361. 10.1007/s00296-010-1478-2 10.1007/s00296-010-1478-2 20401725

[48] Lin C, Chen H-C, Fang W-H, Wang C-C, Peng Y-J et al (2016) Angiotensin-converting enzyme insertion/deletion polymorphism and susceptibility to osteoarthritis of the knee: A case-control study and meta-analysis. PLoS ONE 11:e0161754. 10.1371/journal.pone.016175 27657933 10.1371/journal.pone.0161754 PMC5033346

[49] Ranjbar R, Shafiee M, Hesari A, Ferns GA, Ghasemi F et al (2019) The potential therapeutic use of renin-angiotensin system inhibitors in the treatment of inflammatory diseases. J Cell Physiol 234:2277–2295. 10.1002/jcp.27205 30191985 10.1002/jcp.27205

[50] Chen Y, Sun H, Yao X, Yu Y, Tian T et al (2021) Pharmaceutical therapeutics for articular regeneration and restoration: state-of-the-art technology for screening small molecular drugs. Cell Mol Life Sci 78:8127–8155. 10.1007/s00018-021-03983-8 34783870 10.1007/s00018-021-03983-8 PMC8593173

[51] Tang Y, Hu X, Lu X (2015) Captopril, an angiotensin-converting enzyme inhibitor, possesses chondroprotective efficacy in a rat model of osteoarthritis through suppression local renin-angiotensin system. Int J Clin Exp Med 8:12584–12592 PMID:26550169 26550169 PMC4612854

[52] Yan K, Shen Y (2017) Aliskiren has chondroprotective efficacy in a rat model of osteoarthritis through suppression of the local renin-angiotensin system. Mol Med Rep 16:3965–3973. 10.3892/mmr.2017.7110 28765966 10.3892/mmr.2017.7110 PMC5646976

[53] Wang D, Hu S, Zhu J, Yuan J, Wu J et al (2013) Angiotensin II type 2 receptor correlates with therapeutic effects of Losartan in rats with adjuvant-induced arthritis. J Cell Mol Med 17:1577–1587. 10.1111/jcmm.12128 24112447 10.1111/jcmm.12128 PMC3914644

[54] Habib YH, Sheta E, Khattab M, Gowayed MA (2023) Diminazene aceturate or Losartan ameliorates the functional, radiological and histopathological alterations in knee osteoarthritis rodent model: repurposing of the ACE2/Ang1-7/MasR cascade. J Exp Orthop 10:107–123. 10.1186/s40634-023-00673-1 37878123 10.1186/s40634-023-00673-1 PMC10600085

[55] Ranjit A, Khajeh Pour S, Aghazadeh-Habashi A (2022) Bone-targeted delivery of Novokinin as an alternativetreatment option for rheumatoid arthritis. Pharmaceutics 14:1681. 10.3390/pharmaceutics14081681 36015308 10.3390/pharmaceutics14081681 PMC9416659

[56] Pour SK, Ranjit A, Summerill EL, Aghazadeh-Habashi A (2022) Anti-inflammatory effects of Ang-(1–7) bone-targeting conjugate in an adjuvant-induced arthritisrat model. Pharmaceuticals (Basel) 15:1157. 10.3390/ph15091157 36145378 10.3390/ph15091157 PMC9502795

[57] Soos B, Fagyas M, Horvath A, Vegh E, Pusztai A et al (2022) Angiotensin converting enzyme activity in anti-TNF-treated rheumatoid arthritis and ankylosing spondylitis patients. Front Med (Lausanne) 8:785744. 10.3389/fmed.2021.785744 35155468 10.3389/fmed.2021.785744 PMC8828652

[58] Kacsandi D, Fagyas M, Horvath A, Vegh E, Pusztai A et al (2023) Effect of Tofacitinib therapy on angiotensin converting enzyme activity in rheumatoid arthritis. Front Med (Lausanne) 10:1226760. 10.3389/fmed.2023.1226760 37877017 10.3389/fmed.2023.1226760 PMC10591318

[59] Ytterberg SR, Bhatt DL, Mikuls TR, Koch GG, Fleischmann R et al (2022) Cardiovascular and cancer risk with Tofacitinib in rheumatoid arthritis. N Engl J Med 386:316–326. 10.1056/NEJMoa2109927 35081280 10.1056/NEJMoa2109927

[60] Charles-Schoeman C, Buch MH, Dougados M, Bhatt DL, Giles JT et al (2023) Risk of major adverse cardiovascular events with Tofacitinib versus tumour necrosis factor inhibitors in patients with rheumatoid arthritis with or without a history of atherosclerotic cardiovascular disease: a post hoc analysis from ORAL surveillance. Ann Rheum Dis 82:119–129. 10.1136/ard-2022-222259 36137735 10.1136/ard-2022-222259 PMC9811099

[61] Aymon R, Mongin D, Guemara R, Salis Z, Askling J et al (2025) Incidence of major adverse cardiovascular events in patients with rheumatoid arthritis treated with JAK inhibitors compared with biologic disease-modifying antirheumatic drugs: data from an international collaboration of registries. Arthritis Rheumatol 77:1194–1204. 10.1002/art.43188 40230232 10.1002/art.43188 PMC12401813

[62] Hinkema HJ, Westra J, Arends S, Brouwer E, Mulder DJ (2024) Higher levels of markers for early atherosclerosis in anti-citrullinated protein antibodies positive individuals at risk for RA, a cross sectional study. Rheumatol Int 44:2007–2016. 10.1007/s00296-024-05659-5 39012360 10.1007/s00296-024-05659-5 PMC11393035

[63] Murphy L, Murphy G, Cornally N, McHugh S, Saab MM et al (2025) Low adherence to cardiovascular risk assessment guidelines in patients with rheumatoid arthritis: a retrospective chart review of routine clinical practice. Rheumatol Int 45:158. 10.1007/s00296-025-05916-1 40569458 10.1007/s00296-025-05916-1 PMC12202671

[64] Groten SA, Langerhorst P, Malamas G, Barraclough A, Hoogendijk AJ et al (2025) Targeting synergetic endothelial inflammation by inhibiting NFKB and JAK-STAT pathways. iScience 28:113307. 10.1016/j.isci.2025.113307 40894883 10.1016/j.isci.2025.113307 PMC12396245

[65] Anyfanti P, Angeloudi E, Pagkopoulou E, Boutel M, Moysidou G-S et al (2025) Effects of treatment with Janus kinase inhibitors on coronary microvascular perfusion in patients with rheumatoid arthritis: an observational prospective cohort study. Rheumatol Int 45:111–117. 10.1007/s00296-025-05862-y 40252098 10.1007/s00296-025-05862-y PMC12009227

[66] Seco-Calvo J, Sanchez-Herraez S, Casis L, Valdivia A, Perez-Urzelai I et al (2020) Synovial fluid peptidase activity as a biomarker for knee osteoarthritis clinical progression. Bone Joint Res 9:789–797. 10.1302/2046-3758.911.Bjr-2020-0022.R2 33174472 10.1302/2046-3758.911.BJR-2020-0022.R2 PMC7672324

[67] Ciloglu O, Karaali E, Yilmaz A, Cetinkaya PD, Unlu N et al (2024) Examination of SARS-CoV-2 RNA in joint synovial fluid of patients with COVID-19 and acute knee arthritis. Technol Health Care 32:3793–3800. 10.3233/thc-240317 38788104 10.3233/THC-240317

